# Development of a Ribosomal DNA ITS2 Marker for the Identification of the Thrips, *Scirtothrips dorsalis*

**DOI:** 10.1673/031.010.2601

**Published:** 2010-03-28

**Authors:** R E Farris, R Ruiz-Arce, M Ciomperlik, J D Vasquez, R DeLeón

**Affiliations:** ^1^ United States Department of Agriculture, Center for Plant Health Science and Technology (CPHST), Mission Laboratory 22675 N. Moorefield Rd., Edinburg, TX 78541; ^2^ The Pennsylvania State University, Department of Entomology, 50I ASI Bldg, University Park, PA 16802

**Keywords:** Chilli thrips, invasive pest, PCR primers, molecular diagnostics

## Abstract

The thrips *Scirtothrips dorsalis* Hood (Thysanoptera: Thripidae) is an invasive pest that poses a significant economical threat to U.S. agriculture and trade. In this study, DNA sequence data and polymerase chain reaction (PCR) were utilized to develop a molecular diagnostic marker for *S. dorsalis*. The DNA sequence variation from the internal transcribed spacer 2 (ITS2) region of nuclear ribosomal DNA (rDNA) was analyzed from various thrips species, including *S. dorsalis*. A primer set and polymerase chain reaction cycling parameters were designed for the amplification of a single marker fragment of *S. dorsalis* ITS2 rDNA. Specificity tests performed on ten thrips species, efficacy tests performed on fifteen *S. dorsalis* populations, and tests on primer sensitivity and robustness all demonstrated the diagnostic utility of this marker. This diagnostic PCR assay provides a quick, simple, and reliable molecular technique to be used in the identification of *S. dorsalis*.

## Introduction

There are more than 5,500 species of thrips currently described, and a very small number (1%) are considered to be an agricultural threat (Morse and Hoddle 2006). Of the 100 described species in the genus *Scirtothrips* Shull, ten are considered significant pests of agriculture (Rugman-Jones et al. 2006). *Scirtothrips dorsalis* Hood (Thysanoptera: Thripidae) also known as chilli thrips, yellow tea thrips, castor thrips, assam thrips, and strawberry thrips, is a pest of economic concern with a diverse host range from ornamentals to vegetable and fruit crops (Seal et al. 2005, 2006). Included among the list of hosts, with a potential for heavy crop loss, are chilli pepper, cotton, onion, citrus, banana, bean, peanut, grapes, peach, strawberry, tea, tomato, rose, and various other crops (Ananthakrishnan 1993; CABI/EPPO 1997; Seal et al. 2006). This pest damages its host by feeding on the young leaves, buds, and fruits resulting in feeding scars, color distortions, and stunted growth (Seal et al. 2005, 2006). *Scirtothrips dorsalis* is the only *Scirtothrips* species known to be a vector of certain *Tospoviruses* which include *Groundnut bud necrosis virus*, *Peanut chlorotic fanspot virus*, and *Peanut yellow spot virus* (Whitfield et al. 2005; Meena et al. 2005; Moritz et al. 2000). This species of thrips has a habitat that is widespread ranging from temperate to tropical climatic regions. The geographic distribution of *S. dorsalis* includes Africa, Oceania, and Asia (Seal et al. 2006; Venette and Davis 2003). *Scirtothrips dorsalis* has recently invaded the Caribbean islands of St. Vincent, St. Lucia, and Trinidad (Ciomperlik and Seal 2004). It has also recently been reported from Israel and Kenya (USDA 2005), from Barbados (Ciomperlik et al. 2005 a), Suriname (Ciomperlik et al. 2005), and Puerto Rico (Ciomperlik unpublished data).

This pest species poses a significant economic threat to U.S. agriculture and trade. An estimate of loss can be made: “assuming an overall U.S. crop yield loss from chilli thrips of 10 percent, the total crop value loss would equal $5.98 billion (primary hosts $1.2 billion and secondary hosts $4.78 billion)” (Garrett 2004). Since 1984, USD A-Animal and Plant Health Inspection Service (APHIS) inspectors at various U.S. ports of entry have reported finding live *S. dorsalis* a total of 89 times from imported plant materials of 48 plant taxa (USDA 2003). From 2005 to 2007, it was detected in several counties in the state of Florida and in the southern part of the state of Texas (Ludwig et al. 2007). A suitable climate for *S. dorsalis* is found on approximately 28% of the continental United States (Venette and Davis 2003).

Identification of *S. dorsalis*, as well as other thrips species, has proven difficult due to their minute size and cryptic behavior. Traditional morphological identification of adult specimens requires slide mounting of specimens and knowledge of distinct characters visible through microscopic examination (Palmer et al. 1989; Bisevac 1997). Morphological identification of thrips larvae to the species level is especially difficult and may even be considered impossible (Brunner et al. 2002). Molecular-based methods provide a means for precise identification of difficult to identify species of both adult and immature forms.

Various molecular techniques have been used for thrips species diagnostics as well as for population studies. These have included DNA sequencing (Brunner et al. 2004; Morris and Mound 2004) and PCR-based methods such as real-time PCR (Walsh et al. 2005; Kox et al. 2005), PCR-restriction fragment length polymorphism (PCR-RFLP) (Toda and Komazaki 2002; Brunner et al. 2002; Rugman-Jones et al. 2006), amplified fragment length polymorphism (AFLP) (Fang et al. 2005), and simple sequence repeat (SSR-PCR) (Brunner and Frey 2004). A PCR-RFLP method, developed by Toda and Komazaki (2002), allows nine species of thrips, including *S. dorsalis*, to be identified. This approach has been successfully used for thrips species diagnosis at the CPHST-Mission laboratory (Farris and Ruiz-Arce, unpublished observations). This assay, along with a PCR-RFLP method developed by Rugman-Jones et al. (2006), wherein the identity of seven pest *Scirtothrips* may be determined to species-level, are useful molecular keys in which each thrips species produces unique fragment length polymorphisms.

The accurate detection and identification of pest species is critical to pest management programs. It ensures that the proper control measures are developed for efforts to decrease or eliminate damage posed by unwanted agricultural pests. The objective of this study was to develop a single-step PCR-based marker for the identification of *S. dorsalis*. The development of this molecular-based method would be advantageous by reducing experimental time and monetary costs when compared to other available molecular methods used for these diagnostics. Nuclear ribosomal ITS2 DNA sequence data and PCR methods were applied in the development of this assay. The non-coding ITS2 region, which is a rapidly evolving region, has been widely used for the identification of closely related species (Fritz et al. 1994; Hackett et al. 2000; Torres et al. 2000; Gallego and Galián 2001; Alvarez and Hoy 2002; Thomson et al. 2003; Wilkerson et al. 2004; Naegele et al. 2006). For this PCR assay, primers were designed to amplify a fragment of the ITS2 region from *S. dorsalis* DNA. This body of work results in the development of an assay that produces a single DNA marker fragment. These methods provide a reliable and simple means for identifying individuals belonging to *S. dorsalis*.

## Materials and Methods

### Insect Collection

*Scirtothrips dorsalis* samples were collected from 15 localities representing the Caribbean (N=90), the United States (N=48), India (N=90), Japan (N=90), Thailand (N=30), Suriname (N=30), Venezuela (N=30), and Israel (N=24): where N represents the number of individuals used in this study and does not represent the total number of specimens received, with a few exceptions. Other thrips species submitted for study included *Scirtothrips citri* (Moulton) (CA, USA), *Scirtothrips perseae* Nakahara (CA, USA), *Thrips palmi* Karny (FL, USA), *Thrips tabaci* Lindeman (CA, USA), *Frankliniella occidentalis* (Pergande) (AZ, USA), *Frankliniella shultzei* Trybom (Ethiopia), *Scirtothrips aurantii* Faure (Australia), *Scirtothrips kenyensis* Mound (Kenya), and *Scirtothrips astrictus* Mound and Marullo (Costa Rica). See Table 1 for further information regarding life stage, locality, host, and collection source. Both adult and immature insects were collected either in the field or laboratory from a variety of hosts and were placed in at least 70% ethanol (EtOH). Upon arrival at our laboratory, insect samples were transferred to 90% EtOH and stored at 70° Celsius (C). One to two specimens from each collection were kept as vouchers, and these were stored at -70° C at the USDA CPHST-Mission Laboratory. The names and affiliations of the individuals who contributed to the morphological identification, and collections of thrips housed in the USDA CPHST-Mission Laboratory can be found in the acknowledgements.

### Genomic DNA Isolation

Total genomic DNA was extracted from individual thrips using the DNeasy Blood and Tissue kit (QIAGEN, www.qiagen.com) following the kit protocol “Purification of total DNA from animal tissues”. With the aid of a dissecting microscope, whole individual insects were removed from their vials and placed in 1.5 ml sterile tubes containing lysis buffer (ATL). They were either crushed with a sterile pipet tip in the ATL buffer followed by a 3 hour incubation at 55° C, or they were placed in the ATL buffer overnight at 55° C. The DNA was eluted with a volume of 200 µl sterile water and then concentrated down to 50 µl final volume using a vacufuge instrument (Eppendorf, www.eppendorf.com). For the sensitivity assays, the amount of total genomic DNA in nanograms was quantified using a NanoDrop (ND-1000) spectrophotometer (NanoDrop Technologies, www.nanodrop.com).

**Table 1.**
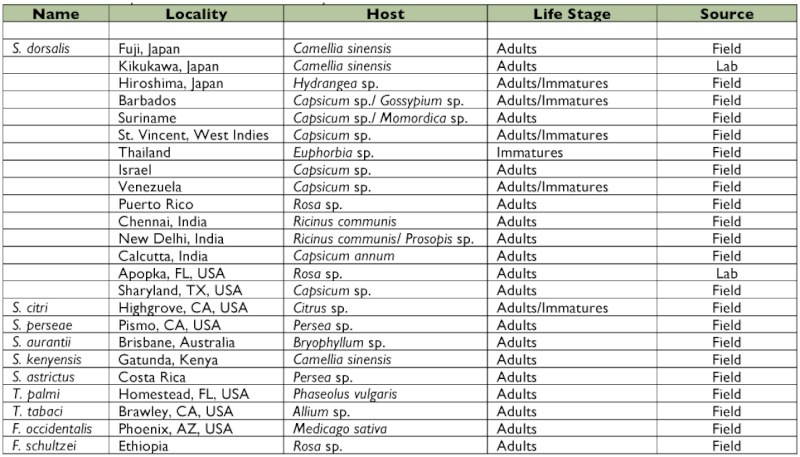
List of Thrips collections used in this study.

### Internal transcribed spacer 2 (ITS2) primer design

Several ITS2 DNA sequences were retrieved from GenBank to aid in designing *S. dorsalis* putative primer pairs. These sequences represented eight thrips species with the following GenBank accession numbers: *F. occidentalis* (AB063334), *F. intonsa* (Trybom) (AB063335), *T. coloratus* Schmutz (AB063338), *T. flavus* Schrank (AB063339), *T. tabaci* (AB063340), *T. palmi* (AB063341), *T. setosus* Moulton (AB063342), and *S. dorsalis* (AB063343) (Toda and Komazaki 2002). Using the Meg Align program (DNASTAR, www.dnastar.com), the DNA sequences were aligned using the ClustalV algorithm (Higgins and Sharp 1988). Regions of sequence variation between these species were targeted as potential primer sites. Primers were designed by eye from the *S. dorsalis* sequence data and then entered into the PrimerSelect program (DNASTAR) to gather information on primer melting temperatures and overall compatability of the putative primer pairs. A total of 28 putative primer pairs were designed to anneal to sequences found within the ITS2 region of *S. dorsalis* DNA. The PCR amplifications for each putative primer pair were carried out using the Takara *Ex Taq* (Hot Start Version) polymerase enzyme (Takara Bio Inc, www.takara-bio.co.jp). The cycling conditions, on a GeneAmp PCR 9700 System thermal cycler (Applied Biosystems, www.appliedbiosystems.com), were optimized for annealing temperature, annealing time, and cycle number. Optimum results, for producing a marker fragment for *S. dorsalis*, occurred with the following primer pair (Operon Biotechnologies www.operon.com): forward (sdi-16F) 5′ GCTTGGATCTGATGGCAAC 3′ and reverse (sdi-20R) 5′ TGTGCACACGAGCCGCTCGC 3′, where the name “sdi” represents *S. dorsalis* ITS2. This primer pair, designed from the ITS2 sequence data of *S. dorsalis* originating from Japan (AB063343), produces a DNA fragment ranging in size from 131 to 135 bp.

### PCR conditions

All sdi-16F/sdi-20R primer tests of specificity, efficacy, and sensitivity were carried out with the following PCR conditions. The PCR amplifications were performed in a final volume of 25 µl using 0.625 units of Takara *Ex Taq* (Hot Start Version) polymerase enzyme (Takara Bio Inc), 2 µl of dNTP mixture (2.5 mM each dNTP), 2.5 µl 10X *Ex Taq* buffer (20 mM MgCl2, 1 µM of forward and reverse primer, and 1 µM of genomic DNA (non-quantified). Negative control reactions were prepared in the same manner but without the addition of genomic DNA. The cycling parameters were carried out on a GeneAmp PCR 9700 System thermal cycler (Applied Biosystems) and were as follows: 1 cycle at 94° C for 3 minutes followed by 26 cycles at 94° C for 30 seconds, 60° C for 10 seconds, 72° C for 1 minute, and ending with an extension cycle of 72° C for 10 minutes.

The PCR products were loaded on 1.2% agarose (A-9539, Sigma-Aldrich, www.sigmaaldrich.com) TBE (Tris/Boric Acid/EDTA) (Bio-Rad Laboratories, www.bio-rad.com) gels pre-stained with ethidium bromide (0.5 ug/ml). Visualization of the amplification products was done using the Flour-S MultiImager system (Bio-Rad Laboratories) and Quantity One analysis software (Bio-Rad Laboratories). The DNA fragment sizes were estimated by comparison to a 100 bp DNA ladder (New England BioLabs, www.neb.com).

### Cloning and DNA Sequencing

To confirm that the developed PCR assay produced an amplicon from the ITS2 region, three PCR products were cloned and sequenced. Samples of *S. dorsalis* DNA from Barbados, India, and Japan were PCR amplified using the sdi-16F/sdi-20R primers. The PCR products were ligated and transformed using the TOPO TA Cloning Kit for Sequencing *with One Shot TOP 10 Electrocomp E. coli* (Invitrogen, www.invitrogen.com). Transformations were plated on LB-kanamycin selective plates. Selected colonies were grown in LB-kanamycin medium overnight at 37° C. Plasmid DNA was extracted from bacterial cultures using the QIAprep Spin Miniprep Kit (QIAGEN). Restriction digests of the plasmid DNA with *Eco*R I followed by agarose gel electrophoresis revealed plasmids that contained the cloned PCR fragment insert. Plasmids positive for the cloned insert were sequenced on the CEQ 8000 Genetic Analysis System (Beckman/Coulter, www.beckmancoulter.com) using the GenomeLab DTCS Quick Start Kit (Beckman/Coulter) and universal primers T3 and T7.

### Tests of Primer Specificity, Efficacy, and Sensitivity

As a measure of quality assurance, the DNA from all thrips samples used in this study was evaluated by performing a PCR assay using thrips primers, designed by Toda and Komazaki (2002), flanking the ITS2 region. Also, all PCR amplifications were performed in duplicate to ensure accurate scoring of the data.

**Figure 1.**
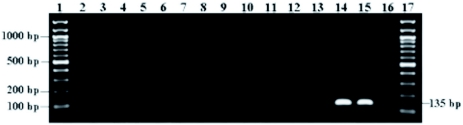
Agarose gel showing PCR results using the ITS2 primer set (sdi-16F/sdi-20R) designed for the detection of *Scirtothrips dorsalis*. Duplicate reactions were performed from single individuals representing seven thrips species. The marker fragment (131–135 bp) was successfully amplified from *S. dorsalis* DNA (lanes 14 and 15), while there was no amplification from the other thrips species (lanes 2 to 13). Lanes 1 and 17, 100 bp DNA ladder; Lanes 2 and 3, *T. palmi*; Lanes 4 and 5, *T. tabaci*; Lanes 6 and 7, S. citri; Lanes 8 and 9, *F. occidentalis*; Lanes 10 and 11, *S. perseae*; Lanes 12 and 13, *F. schultzei*; and Lane 16, negative control. High quality figures are available online.

**Figure 2.**
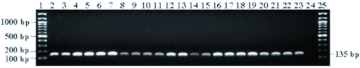
Agarose gel showing the PCR amplification of the ITS2 diagnostic marker for *Scirtothrips dorsalis*. Duplicate reactions were performed from single individuals representing 11 *S. dorsalis* populations. Lanes 1 and 25, 100 bp DNA ladder; Lanes 2 and 3, Fuji, Japan; Lanes 4 and 5, Kikukawa, Japan; Lanes 6 and 7, Hiroshima, Japan; Lanes 8 and 9, Barbados; Lanes 10 and 11, Suriname; Lanes 12 and 13, St. Vincent; Lanes 14 and 15, Thailand; Lanes 16 and 17, Israel; Lanes 18 and 19, Venezuela; Lanes 20 and 21, Puerto Rico; Lanes 22 and 23, New Delhi, India; and Lane 24, negative control. High quality figures are available online.

The specificity of the sdi-16F/sdi-20R *S. dorsalis* primer pair was evaluated by performing PCR assays on ten thrips species. This included 30 individual specimens from each of *T. palmi*, *T. tabaci*, *S. citri*, *S. perseae*, and *F. occidentalis* and 22 individual specimens of *S. astrictus*, 5 of *F. schultzei* and *S. aurantii*, and 1 of *S. kenyensis* (Table 2, Figure 1). *Scirtothrips dorsalis* DNA was used as a positive control to determine that each amplification of the marker was successful. To determine the efficacy of the primer set, a total of 432 *S. dorsalis* specimens representing 15 geographic populations were assayed (Table 3, Figure 2).

**Table 2.**
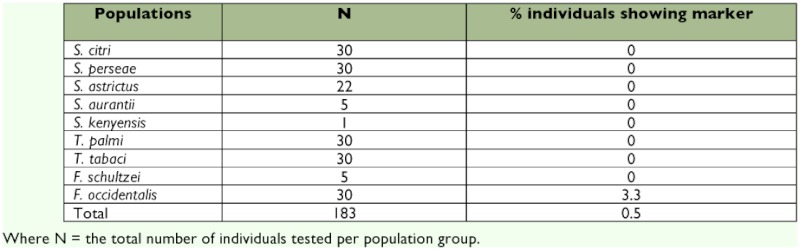
*S. dorsalis* ITS2 marker data for thrips species.

**Figure 3.**
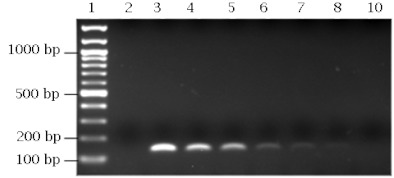
PCR amplification of the *Scirtothrips dorsalis* marker using a DNA template dilution series. Lane 1, 100 bp DNA ladder; Lane 2, negative control; and Lanes 3 to 9, include amplification products from the following DNA amounts; 12 ng, 6 ng, 3 ng, 1 ng, 0.5 ng, 0.25, and 0.1 ng, respectively. High quality figures are available online.

**Figure 4.**
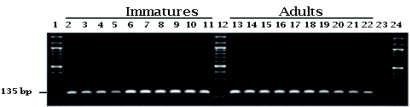
Five Scirtothrips *dorsalis* individuals from each of two life stages were amplified with the ITS2 primer set (sdi-16F/sdi-20R). Lanes 1, 12, and 24, 100 bp DNA ladder; Lanes 2 to 11, immatures; Lanes 13 to 22, adults; and Lane 23, negative control. High quality figures are available online.

To further evaluate the *S. dorsalis* primer pair, tests were performed to examine the sensitivity of the assay. To determine the lowest amount of *S. dorsalis* template DNA detectable with this PCR assay, A DNA template dilution series was performed on seven samples representing populations from India (1), Suriname (1), and Barbados (5). The DNA templates were prepared at 12, 6, 3, 1, 0.5, 0.25, 0.1 and 0.05 ng total DNA and tested (Figure 3). Tests comparing *S. dorsalis* adults and immatures (Figure 4) and *S. dorsalis* males and females were also performed.

**Table 3.**
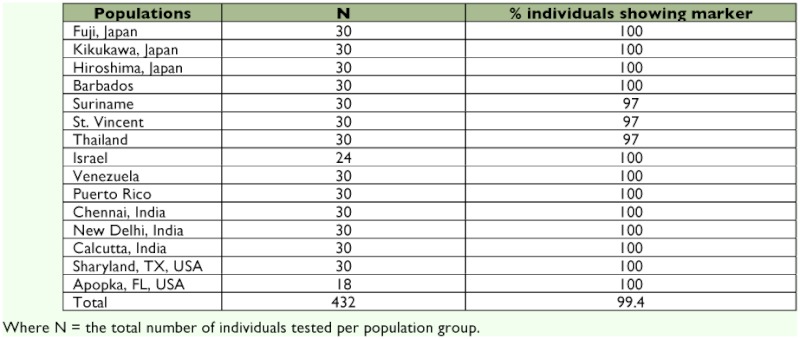
*S. dorsalis* ITS2 marker data for *S. dorsalis* populations.

### Robustness Test

To evaluate the robustness of this diagnostic primer pair, alternative PCR conditions and cycling parameters were tested on a smaller scale where five individuals from each *S. dorsalis* population and thrips species, with the exception of *S. kenyensis*, were tested. To test robustness, OmniMix HS Lyophilized universal PCR reagent beads (Cepheid, www.cepheid.com), which contain all the necessary PCR components, were used in the PCR reactions following the manufacturer's instructions with a 1 µM final concentration of each primer and 1 µl of genomic DNA (non-quantified). The cycling parameters for these reactions were carried out on the SmartCycler System (Cepheid) as follows: 1 cycle at 95° C for 2 minutes followed by 30 cycles at 95° C for 15 seconds, 58°C for 10 seconds, 72°C for 30 seconds, and ending with an extension cycle of 72° C for 2 minutes.

## Results and Discussion

### ITS2 Sequence Analysis and assay design

Alignment of the ITS2 sequences from the 8 thrips species, obtained through GenBank, revealed variation among all species. Notably, since the focus was to develop PCR primers that are specific to *S. dorsalis*, the DNA sequence regions in which *S. dorsalis* was most dissimilar, compared to the other species, were targeted. A total of 19 primers were identified by eye. Various primer pair combinations were tested with the PrimerSelect program that provides information on primer-dimers, hairpins, product length, and annealing temperature. A total of 28 putative *S. dorsalis* primer pairs were obtained. Initial screening of these primer pairs, using *S. dorsalis* DNA, showed that most pairs did not produce their expected fragment: some pairs produced no DNA fragment, and other pairs produced various DNA fragments. While optimization of the assay conditions on these unsuccessful primer pairs may have improved the results, it was not done, since other primer pairs were shown to be successful.

**Figure 5.**
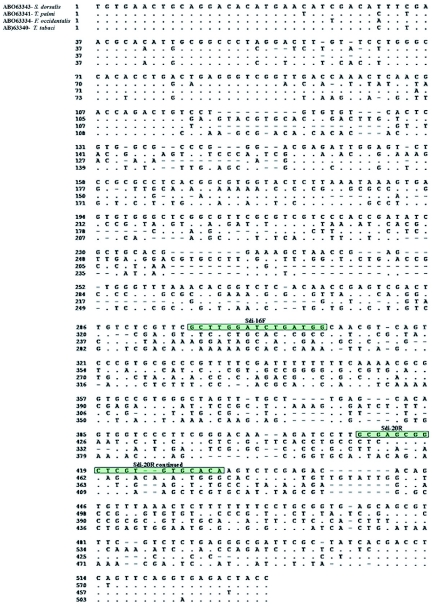
Clustal V alignment of the ITS2 region from Scirtothrips *dorsalis* and other thrips species. The sdi-16F and sdi-20R primer sequences are highlighted. Dots indicate sequence similarity to the *S. dorsalis* sequence. Dashes (-) indicate gaps showing insertion/deletions. High quality figures are available online.

Of the 28 primer pairs screened, eight produced a single amplicon of expected size. Evaluation of the DNA sequences between these eight primer pairs was performed to explore further regions of variability that may be useful for future developmental work, and this led to the selection of four primer pairs. As a preliminary test for specificity, these four primer pairs were screened for cross reactivity with the following thrips species: *T. tabaci*, *T. palmi*, *F. occidentalis*, *F. schultzei*, *S. citri*, and *S. perseae*. The best results, with respect to PCR efficacy and specificity to *S. dorsalis,* were produced with the sdi-16F/sdi-20R primer pair. Figure 5 shows a clustal V alignment of the ITS2 region from four of the eight thrips species used to design the primers; the sdi-16F/sdi-20R primer sequences are noted on the *S. dorsalis* sequence. As can be seen in Figure 5, there is little homology between *S. dorsalis* and the other thrips species.

A BLAST search analysis (National Center for Biotechnology, http://www.ncbi.nlm.nih.gov/) was performed to find regions of sequence similarity between database sequences and the sdi-16F/sdi-20R primer sequences. This search provided information on the specificity of the primer sequences for *S. dorsalis*. The sdi-16F primer sequence produced alignments with 6 *Scirtothrips* species in addition to *S. dorsalis*, and these included *S. pan*, *S. perseae*, *S. aceri*, *S. astrictus*, *S. oligochaetus*, and *S. n. sp*. The sequences of these 6 species were obtained and aligned with the *S. dorsalis* marker sequence to evaluate percent identity. An average of 55% identity was observed between *S. dorsalis* and *S. perseae*, *S. aceri*, *S. astrictus*, *and S. n. sp*, while there was a 66% identity with *S. pan* and an 89% identity with *S. oligochaetus*. The sdi-20R primer sequence produced a significiant alignment with the single thrips species, *S. dorsalis*. Therefore, BLAST data shows that these primers, in combination, should produce an *S. dorsalis* marker, and it also supports that the specificity of the primer pair is due in large part to the sdi-20R primer.

To confirm the specified region of the *S. dorsalis* amplicon, PCR products representing *S. dorsalis* from Barbados, India and Japan were cloned, sequenced and submitted for BLAST analysis. Sequence analysis of the cloned PCR products revealed that the *S. dorsalis* amplicon ranged in size from 131 to 135 bp with India producing a 131 bp fragment, Barbados producing a 132 bp fragment, and Japan producing a 135 bp fragment (GenBank accession numbers: FJ168682 - FJ168684). The DNA sequence data from the cloned *S. dorsalis* Japan sample (FJ168684) was identical to the Japan *S. dorsalis* sequence (AB063343) used in the design of the sdi-16F/sdi-20R primer-pair. The BLAST results revealed significant homology between the 131–135 bp fragment sequences and *S. dorsalis* ribosomal DNA. The significant alignments produced between these sequences and ribosomal sequence data supports that this amplicon is representative of the ITS2 region.

### Primer Specificity, Efficacy and Sensitivity

To confirm the diagnostic utility of the developed PCR assay, tests for specificity, efficacy, and sensitivity were performed. The specificity of the sdi-16F/sdi-20R *S. dorsalis* primer pair was evaluated by performing PCR assays on a total of 183 individual thrips, and this included 1 to 30 individual specimens from each of ten thrips species including *S. dorsalis* (Table 2, Figure 1). For *S. citri*, *S. perseae*, *S. astrictus*, *S. aurantii*, *S. kenyensis*, *T. palmi*, *T. tabaci*, *and F. schultzei* no cross-reactivity was observed. However, for *F. occidentalis*, one out of the 30 individuals tested for this species showed amplification of the marker fragment, but it appeared very faint and distinctly different from the positive control that displayed ample amplification of the marker fragment. Therefore, only one out of a total of 183 individual thrips or 0.5 % showed amplification of the marker band. It is important to note that the *F. occidentalis* species can be visually distinguished from the *S. dorsalis* species at the adult stage by using overall size and color as well as standard morphological characters. We recognize that not all *Scirtothrips* species, pestiferous and non-pestiferous, were represented in this study. However, of the ten pest *Scirtothrips* species, six were included in this study. Pest *Scirtothrips* that may be encountered in the U.S. include *S. aceri*, *S. citri*, *S. perseae*, and *S. dorsalis* (Hoddle et al. 2008). Also, we recognize the small sample size for some species, such as *S. kenyensis*, screened with this assay. Testing this assay on greater numbers of these species as well as on additional species, such as *S. aceri*, is important and will provide a larger data set as a reference for this diagnostic tool.

The results of the efficacy tests done on 432 *S. dorsalis* specimens representing 15 geographic populations showed 12 populations that displayed 100%) amplification of the expected marker fragment, and these included the populations from India, Japan, the U.S., Barbados, Israel, and Venezuela (Table 3, Figure 2). For the populations from St. Vincent, Suriname, and Thailand, 29 out of 30 individuals or 97% showed amplification of the marker fragment (Table 3). Overall, 429 or over 99% of the *S. dorsalis* individuals displayed the expected marker amplicon ranging in size from 131 to 135 bp. Amplification of this DNA marker from a wide geographic representation of *S. dorsalis* samples supports the robustness of this PCR assay. Previous molecular data has suggested that *S. dorsalis* may be comprised of several species (Hoddle et al. 2008; Rugman-Jones et al. 2006). Rugman-Jones et al (2006) concluded that *S. dorsalis* from India and South Africa were different species, based on their PCR-RFLP results. The phylogenetic analysis performed by Hoddle et al (2008) shows evidence that *S. dorsalis* separates into at least 3 groups. Of the *S. dorsalis* populations used in their studies, we lacked those from Australia, Africa, and Taiwan. Testing this assay on more geographic populations of *S. dorsalis*, such as those from Australia and Africa, will enable us to expand our dataset and provide further insight into the *S. dorsalis* complex.

Due to the small size of the insect and the fact that sometimes only insect partials may be obtained for analysis, it was important to study the analytical sensitivity of this assay. This was done by determining the lowest concentration of *S. dorsalis* template DNA detectable with this PCR assay. Primer sensitivity to various concentrations of *S. dorsalis* DNA template (0.05 to 12 ng) from seven individuals showed that, on average, the detection limit of total thrip DNA was 0.25 ng (Figure 3). The detection limit observed from a single DNA template (India) was 0.05 ng. In a test comparing two life stages, the *S. dorsalis* marker was produced from both immature and adult DNA templates indicating that life stage does not appear to be a factor on quality or sensitivity of these primers (Figure 4). Amplification of DNA from *S. dorsalis* males and females was also compared. The results revealed no difference in amplification of the marker fragment from any of the individuals examined.

To further explore the robustness of the sdi-16F/sdi-20R primer set, different assay conditions were tested on a sub-group of samples representing all fifteen *S. dorsalis* populations and nine thrips species. The PCR reactions were prepared with OmniMix HS Lyophilized universal PCR reagent beads (Cepheid) and cycling conditions were performed on the SmartCycler System (Cepheid). All 75 or 100% of the *S. dorsalis* individuals tested (5 per geographic population) produced the expected marker fragment. Of the 41 individuals tested from the remaining thrips species (5 per species and 1 from *S. kenysensis*), none of them produced the marker fragment. The 100%) agreement between these results and the results obtained using different PCR reagents and platforms demonstrates the robustness of this primer set for diagnosis. Therefore, either of the two PCR conditions (Ex-taq/9700 or Omni/SmartCycler) may be used for the detection of *S. dorsalis* DNA.

In conclusion, the molecular data gathered from tests for specificity, efficacy, sensitivity, and robustness reveal that this newly developed single PCR assay, producing a 131–135 bp ITS2 marker fragment, can serve as a valuable diagnostic tool for the identification of *S. dorsalis*. Compared to PCR-RFLP methods that require PCR followed by a restriction digest such as those designed by Rugman-Jones et al (2006) and Toda and Komazaki (2002), this method requires a single PCR step. These PCR-RFLP methods provide keys to identify several thrips species, whereas our method identifies only the *S. dorsalis* species. There are several advantages of this single-step PCR method. Results may be obtained faster than most other molecular methods. It is more cost effective, since it eliminates the use of restriction enzymes, eliminates the need to prepare additional agarose gels, and it saves on time. Interpretation of the results is simple, since the presence of the marker band versus the non-presence of the band is sufficient to provide a diagnostic for the *S. dorsalis* species. The robustness of this diagnostic tool, demonstrated in this study, should allow it to be easily transferred to other molecular laboratories.

## Abbreviation

ITS2: Internal transcribed spacer 2
